# Association Between Lipoprotein(a) and Arterial Stiffness in Young Adults with Familial Hypercholesterolemia

**DOI:** 10.3390/jcm14051611

**Published:** 2025-02-27

**Authors:** Sibbeliene E. van den Bosch, Lotte M. de Boer, Alma Revers, Eric M. Schrauben, Pim van Ooij, Aart J. Nederveen, Willemijn E. Corpeleijn, John J.P. Kastelein, Albert Wiegman, Barbara A. Hutten

**Affiliations:** 1 Department of Pediatrics, Amsterdam University Medical Center, Location AMC, 1105 AZ Amsterdam, The Netherlands; 2 Department of Epidemiology and Data Science, Amsterdam University Medical Center, Location AMC, 1105 AZ Amsterdam, The Netherlands; 3 Amsterdam Gastroenterology Endocrinology Metabolism Research Institute, 1105 AZ Amsterdam, The Netherlands; 4 Amsterdam Cardiovascular Sciences Research Institute, Diabetes & Metabolism, 1105 AZ Amsterdam, The Netherlands; 5 Department of Radiology and Nuclear Medicine, Amsterdam University Medical Center, location AMC, 1105 AZ Amsterdam, The Netherlands; 6 Department of Vascular Medicine, Amsterdam University Medical Center, location AMC, 1105 AZ Amsterdam, The Netherlands

**Keywords:** lipoprotein(a), familial hypercholesterolemia, atherosclerosis, arterial stiffness, pulse wave velocity

## Abstract

**Background and Aims**: Elevated lipoprotein(a) [Lp(a)] and familial hypercholesterolemia (FH) are both inherited dyslipidemias that are independently associated with cardiovascular disease. Surrogate markers to assess signs of atherosclerosis, such as arterial stiffness, might be useful to evaluate the cardiovascular risk in young patients. The aim of this study is to evaluate the contribution of Lp(a) to arterial stiffness, as measured by carotid pulse wave velocity (cPWV) in young adults with FH. **Methods**: For this cross-sectional study, 214 children with FH who participated in a randomized controlled trial between 1997 and 1999 on the efficacy and safety of pravastatin were eligible. After 20 years, these patients were invited for a hospital visit, including cPWV assessment (by 4D flow MRI) and Lp(a) measurement. Linear mixed-effects models were used to evaluate the association between Lp(a) and cPWV. **Results**: We included 143 patients (mean [standard deviation] age: 31.8 [3.2] years) from 108 families. Median (interquartile range) cPWV was 1.62 (1.31–2.06) m/s. Both the unadjusted (ß = −0.0014 m/s per 1 mg/dL increase in Lp(a), 95% CI: −0.0052 to 0.0023, *p* = 0.455) and adjusted model (ß = −0.0005 m/s per 1 mg/dL increase in Lp(a), 95% CI: −0.0042 to 0.0032, *p* = 0.785) showed no significant association between Lp(a) and cPWV. **Conclusions**: Our findings indicate that Lp(a) levels are not associated with carotid arterial stiffness in young adults with FH. Possibly, High Lp(a) might cause atherosclerosis by mechanisms beyond arterial stiffness in young adults. Other surrogate markers of early signs of atherosclerosis may be more suitable to evaluate the Lp(a)-mediated contribution to atherosclerosis in young FH patients.

## 1. Introduction

Elevated levels of lipoprotein(a) [Lp(a)] and familial hypercholesterolemia (FH) are both inherited dyslipidemias that are independently associated with atherosclerotic cardiovascular disease (ASCVD). Several studies in middle-aged adults have demonstrated that when both risk factors are present, the ASCVD risk is even higher due to lifelong exposure to these two genetic risk factors [1,2,3,4]. However, the pathophysiological role of Lp(a) in this elevated risk is not fully understood. Given that children and young adults with both FH and high Lp(a) levels remain clinically silent until later in life, the use of surrogate markers to assess early signs of atherosclerosis is of great importance in evaluating the ASCVD risk in young patients. A recent study in children with FH that were followed-up for nearly 20 years showed that higher Lp(a) levels contributed significantly to arterial wall thickening. This suggests that elevated Lp(a) might be an additional and independent risk factor for atherosclerosis in young FH patients [5]. Apart from wall thickening, Lp(a) is involved in atherosclerosis by inflammatory and prothrombotic mechanisms. This indicates a potential effect on arterial stiffness, directly associated with ASCVD, measured by pulse wave velocity (PWV) [6,7]. PWV can be measured through methods, such as tonometry or magnetic resonance imaging (MRI), including 4-dimensional (4D) flow. The 4D flow technique is acquired with a high spatial resolution, which allows for blood flow to be measured at approximately 110–150 unique locations along the length of the common and internal carotid arteries. Therefore, the use of this modern and advanced technique will enable us to identify more premature phases of atherosclerosis than other methods [8,9,10,11]. Although a recent Mendelian randomization study in a population-based cohort showed no association between Lp(a) and arterial stiffness, as measured by brachial-ankle and carotid-femoral PWV, it is possible that 4D flow MRI provides a more accurate assessment of carotid PWV (cPWV), particularly in relatively young patients with FH, where signs of atherosclerosis are often less pronounced [12].

Given the association between lipoprotein metabolism and arterial changes, we hypothesize that arterial stiffness is higher in young patients with FH and elevated levels of Lp(a). To the best of our knowledge, no studies have specifically investigated the role of Lp(a) in arterial stiffness within the context of FH patients. Therefore, the aim of this study is to evaluate the association between Lp(a) and cPWV in young adults with FH using the advanced and modern 4D flow MRI technique.

## 2. Methods

### 2.1. Study Design and Study Population

For this cross-sectional study, all 214 children (aged 8–18 years) with heterozygous FH who had undergone randomization from 1997 to 1999 in a single-center, double-blind, placebo-controlled trial on the efficacy and safety of pravastatin, were eligible. After trial participation, statin therapy was initiated or continued. All children were genetically tested, and 210 (98%) had a documented pathogenic mutation in the genes encoding LDL receptor or apolipoprotein B [13]. Approximately 20 years after randomization (i.e., 2015–2017), these patients were invited for a hospital visit, as described previously [14]. During this visit, information on medical history, lifestyle habits, medication use, and family history was obtained; a physical examination was performed, and blood samples were taken. In addition, a 4D flow MRI scan covering the carotid arteries of the neck was performed. For the current study, patients were included if both a cPWV measurement and an Lp(a) measurement were available.

This study complies with the Declaration of Helsinki. The study protocol was approved by the Institutional Review Board at the University of Amsterdam. All patients provided written informed consent.

### 2.2. Lipids and Lipoproteins

In the fasted blood sample, total cholesterol, high-density lipoprotein cholesterol (HDL-C) and triglyceride levels were determined by commercially available kits (Cobas c502 and c702 chemical analyzers, Roche Diagnostics). The level of LDL-C was calculated with the Friedewald equation [15]. Lp(a) levels were freshly analyzed in the clinical laboratory of the Amsterdam University Medical Centers—Location AMC. Lp(a) levels were determined by an immunoturbidimetric (mass) assay (Quantia Lp(a), Architect C-8000 system, Abbott). This assay is calibrated for 5 points of apo(a) isoforms to minimize isoform sensitivity. In case of a result above the upper limit of the measuring range, the sample was diluted. To correct the LDL-C level (LDL_cor_-C) for Lp(a)-cholesterol (Lp(a)-C), we assumed that 30% of the Lp(a) mass consists of Lp(a)-C.

### 2.3. Pulse Wave Velocity of the Carotid Artery

4D flow MRI is a technique that measures 3D blood flow as a function of time (4D), resulting in volumetric flow quantification and visualization. We used the following scan parameters for 4D flow MRI to determine the cPWV measurements: TR/TE = 7.99/4.55 milliseconds (ms), flip angle 8°, velocity encoding = 150 cm/second (cm/s), spatial resolution = 0.8 × 0.8 × 0.8 mm^3^ and acquired temporal resolution ≈ 80 ms. Cardiac synchronization was performed using retrospective ECG triggering.

**Figure 1:**
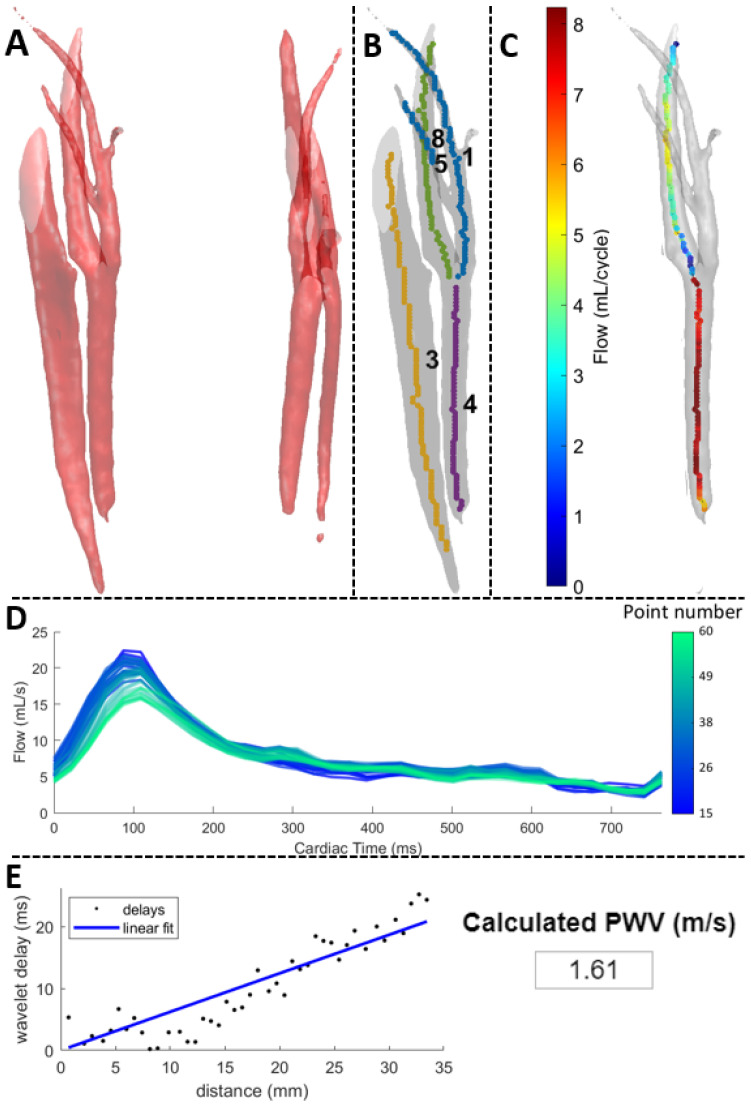
Illustration of the measurement of the carotid pulse wave velocity in a single patient using the open-source Amsterdam UMC FlowProcessingTool Matlab-based app (Matlab^®^ version 2021a) (11, 16). (**A**) 3D segmentation of the vessels in the neck. (**B**) Identification of right carotid with corresponding centerlines. (**C**) Total flow (mL/cycle) visualization in the right carotid. (**D**) Flow waveforms (mL/s) along corresponding points, with clear delays in more distal points. (**E**) Calculation of carotid PWV (m/s), using a linear fit to distance versus waveform delays. PWV: pulse wave velocity.

To calculate the cPWV (meter/second [m/s]) of both carotids, we used the open-source Amsterdam UMC FlowProcessingTool Matlab-based app (Matlab^®^ version 2021a) developed by Schrauben et al. [11,16] (Figure 1). 4D flow MRI data were further reconstructed to facilitate the use of the FlowProcessingTool; images were reconstructed to a temporal resolution of approximately 25 ms. The FlowProcessingTool automatically creates a 3D segmentation view of the neck vessels using the 4D flow-derived angiographic image (Figure 1A). Following automatic centerline extraction from this segmentation (Figure 1B), we manually identified the common carotid, the carotid bulb, and the internal carotid segments on both the left and right carotid arteries choosing the corresponding anatomical measurement points provided by the tool. This step was performed by one researcher and verified by a second researcher. During the data extraction on cPWV using the FlowProcessingTool, the researchers were blinded to all other patient characteristics. At each centerline point within the artery, blood flow is automatically measured and plotted (Figure 1C,D). cPWV is then calculated as the linear fit of distance along the artery versus the delays between successive flow waveforms [17] (Figure 1E). As the main outcome measurement we used the mean cPWV (m/s). Mean cPWV was defined as the mean PWV of the right and the left carotid artery. In addition, we assessed cPWV per segment, i.e., the common carotid, the carotid bulb, and the internal carotid segments. For each given segment, cPWV was defined as the average of the right and left PWV measurements. If on either side a segment was missing, cPWV was defined as the value of the remaining segment; if both left- and right-side values were unavailable, the cPWV value was considered missing for that segment.

### 2.4. Statistical Analysis

Linear mixed effects models with a random intercept per family were used to evaluate the association between Lp(a) (both as a continuous variable and dichotomized to low and high [below or above 50 mg/dL [18]]) and mean cPWV. In addition, despite the highly skewed distribution of Lp(a) levels, we assumed linearity between the independent variable Lp(a) levels and dependent variable cPWV based on an evaluation of model fit using visual evaluation, AICs, and the ANOVA test. For the univariable associations between the variables and the dependent variable Lp(a), the Lp(a) was log transformed. Based on literature and clinical relevance, we considered the following variables as potential confounders: age, sex, body mass index (BMI), mean arterial pressure (MAP), smoking status, high-density lipoprotein cholesterol (HDL-C), LDL-C, triglycerides and statin use [6]. Variables were added as potential confounders in the full model based on the univariable association between either Lp(a) and cPWV. A variable with a *p*-value < 0.4 in at least one of the two associations was included in the full model. After this, we created a final model by considering every combination of potential confounders while retaining the determinant Lp(a). The final model had the lowest Akaike information criterion.

As a sensitivity analysis, we also performed the analyses with LDL-C not being corrected for Lp(a)-C (LDL-C) and with Lp(a)-C being estimated as 15% and 45% of the Lp(a) mass (LDL_cor15%_-C and LDL_cor45%_-C), respectively. In addition, all analyses were repeated for each segment of the carotid artery (common carotid, carotid bulb, and internal carotid), separately.

*p*-values are two-sided and were considered statistically significant if <0.05. All analyses were performed using the R statistical package, version v.4.2.1 (R foundation for Statistical Computing; Vienna, Austria).

## 3. Results

### 3.1. Description of the Study Population

In 143 patients from 108 families, both a cPWV measurement and Lp(a) level were available and they comprised our study population. Reasons for non-participation in the MRI part of the study were not willing/no time (n = 15), claustrophobia (n = 7), potential pregnancy (n = 2), presence of metal splinters (n = 1) or no availability of MRI scan or technical issues during the visit (n = 16). Demographic and clinical characteristics of these patients are displayed in Table 1. Mean (SD) age was 31.8 (3.2) years and almost half of them were male (48.3%). Mean (SD) LDL-C and LDL_cor30%_-C were 4.1 (1.7) and 3.9 (1.8) mmol/L, respectively. The great majority of patients used statins (80.4%) and in 28 (19.6%) patients, LDL-C levels were below 2.5 mmol/L, the target LDL-C value for adult patients with FH. The median (IQR) Lp(a) level was 12.8 (5.4–24.34) mg/dL and in 22 (15.4%) of the patients, Lp(a) levels exceeded 50 mg/dL.

### 3.2. Association Between Lipoprotein(a) and Pulse Wave Velocity

Median (IQR) cPWV was 1.62 (1.31–2.06) m/s. Results of the unadjusted analysis showed no significant association between Lp(a) and mean cPWV (ß = −0.0014 m/s per 1 mg/dL increase in Lp(a), 95% CI: −0.0052 to 0.0023, *p* = 0.455). Based on the univariable association between either Lp(a) or mean cPWV, the following patient characteristics were selected as potential confounders and included in the full model (Supplementary Tables S1 and S2): age, sex, BMI, LDL_cor30%_-C, triglycerides, HDL-C, statin use after 20 years of age and MAP. After model selection, sex, BMI, LDL_cor30%_-C and MAP remained as potential confounders in the model (Table 2, final model; Supplementary Table S2). Similar to the unadjusted model, the results of this model showed no significant association between Lp(a) and mean cPWV (ß = −0.0005 m/s per 1 mg/dL increase in Lp(a), 95% CI: −0.0042 to 0.0032, *p* = 0.785).

**Figure 2:**
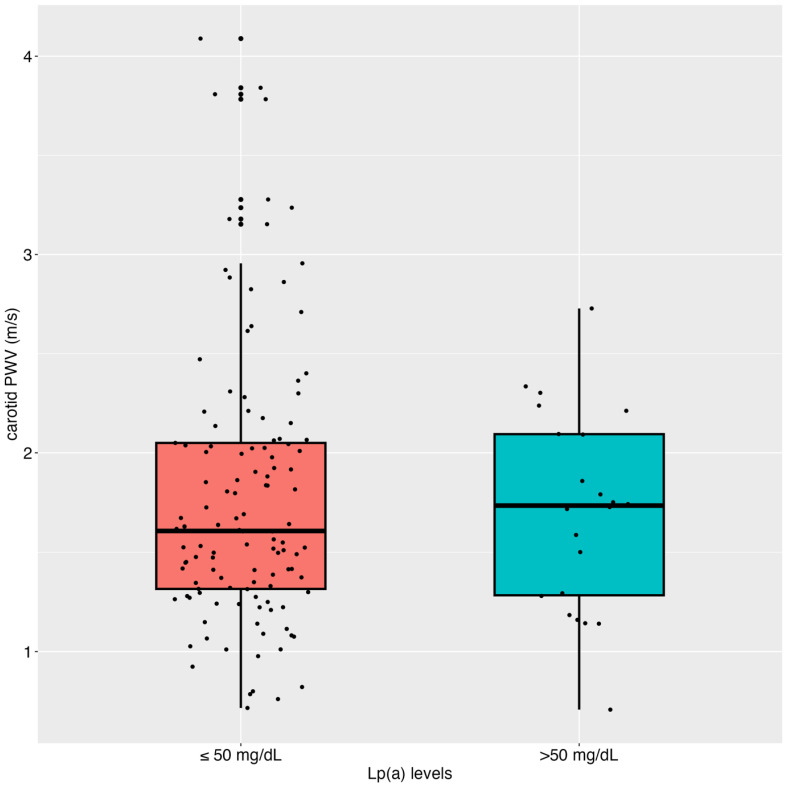
Median lipoprotein(a) levels in patients with levels below and above 50 mg/dL. PWV: pulse wave velocity; m/s: meter per second; mg/dL: milligram per deciliter.

Figure 2 displays the median (IQR) cPWV for Lp(a) levels below and above 50 mg/dL. Median (IQR) cPWV levels did not differ significantly among patients with low and high Lp(a) levels (1.61 [1.32 to 2.05] m/s vs. 1.73 [1.28 to 2.09] m/s, *p* = 0.922. When repeating the linear mixed model analysis after dichotomizing Lp(a) levels to below and above 50 mg/dL, no significant associations (unadjusted: *p* = 0.626 and adjusted: *p* = 0. 999) between Lp(a) and mean cPWV were found. When including LDL-C, LDL_cor15%_ or LDL_45%_-C in the models instead of LDL_cor30%,_ results were comparable to the models, including LDL_cor30%_.

We also performed the analyses for each carotid segment (the common carotid artery, carotid bulb, and the internal carotid artery) separately, and the results were comparable to the models including the whole carotid artery.

## 4. Discussion

In the current study, we evaluated the association between Lp(a) and arterial stiffness, measured by 4D flow cPWV, in young adults with FH. We found no significant association between Lp(a), both as a continuous and as a dichotomous variable, and arterial stiffness, suggesting that Lp(a) might not contribute to arterial stiffness in young adults with FH.

In the past, several studies evaluated arterial stiffness in young FH patients. However, most studies did not include Lp(a) levels and/or used different locations and techniques to assess PWV [19]. Similar to our findings, a study in children (n = 257) with a positive family history for CVD and high (Lp(a) ≥ 30 mg/dL, 42.8%) vs. normal (Lp(a) < 30 mg/dL, 57.2%) Lp(a) levels showed no significant differences in PWV (assessed using tonometry) between these two groups [20]. In addition, investigators of a recent Mendelian randomization study and a recent cross-sectional study did not find evidence supporting a causal association between Lp(a) and PWV in the general population [12,21]. Results of other studies on the association between Lp(a) and arterial stiffness are inconsistent. However, as these studies were conducted in older patients with comorbidities (such as hypertension, diabetes, and renal failure), the results of these studies are not directly comparable to the results of our study [22,23,24,25]. Previous studies showed that different risk factors are related to arterial stiffness, including age [26]. Our study population is relatively young and has been receiving lipid-lowering therapy from an early age to normalize their cholesterol levels. Our cohort may have been too young and the age range too small—potentially leading to a small range of cPWV—of these otherwise healthy young adults, which may have contributed to the absence of an observed association between Lp(a) and arterial stiffness.

In recent years, growing evidence elucidated the role of Lp(a) in the development of atherosclerosis [27]. Like LDL-C, Lp(a) invades the arterial wall and triggers inflammatory and oxidative processes, which have atherothrombotic effects on the artery walls [28]. Lp(a) also induces endothelial dysfunction by upregulating adhesion molecules like VCAM-1 and E-selectin, promoting atherosclerosis [28]. Additionally, Lp(a) carries oxidized phospholipids that attach to monocytes, leading to atherosclerosis and arterial wall inflammation [28]. One could speculate that high Lp(a) levels might induce arterial stiffness as a result of this cascade. The fact that studies in older adults with other cardiovascular risk factors, such as hypertension, showed an association between Lp(a) and arterial stiffness underlines this hypothesis. However, experimental evidence suggesting a positive association between Lp(a) and arterial stiffness is scarce and also the results of the current study in young FH patients do not substantiate this [23,29,30].

In children, flow-mediated vasodilatation (FMD) is a sensitive surrogate marker for endothelial dysfunction by atherosclerosis, and it has been observed to be elevated in children with elevated Lp(a) levels [31,32]. Although (subclinical) atherosclerosis and arterial stiffness are frequently detected in conjunction, the relationship between these processes has not been thoroughly investigated, particularly not in young adults. Based on our results, one could hypothesize that Lp(a)-mediated arterial stiffness may only become significant at an older age, or that other markers of atherosclerosis might be better at detecting early signs of atherosclerosis in young individuals. Accordingly, in a recent study of the current cohort, a positive association was found between Lp(a) and carotid intima-media thickness (cIMT) during a 20-year follow-up in FH patients, supporting the aforementioned hypothesis [5]. As we did not find an association between Lp(a) and arterial stiffness in this study, it may be possible that Lp(a) mediates atherosclerosis through mechanisms beyond arterial stiffness. In addition, we did not find a significant association between LDL-C and cPWV either, suggesting that ApoB-containing lipoproteins, such as LDL-C and Lp(a), do not mediate arterial stiffness at this young age.

Several limitations need to be considered in this study. First, due to its observational nature, we adjusted the analysis for known confounders as far as possible. However, some confounders may not be assessed accurately, and/or other confounders, which remain unknown to date, are not assessed at all. Therefore, the association between lipoprotein(a) and cPWV might have been obscured by residual or unmeasured confounding. Second, we did not observe a statistically significant association between Lp(a) and cPWV. However, it is possible that the size of our study population was insufficient to detect an association that may genuinely exist. Third, we combined the cPWV of the left and right (segments of the) carotid artery, assuming that these would not differ. However, as a result of different anatomical origins, flow in the right segments might be more turbulent and more prone to arterial stiffness, and thus, these two flow measurements cannot be combined [33]. However, we deem that the impact of these potential (small) differences between the left- and right arteries will be marginal for the association between Lp(a) and cPWV. Fourth, the random intercepts per family were difficult to identify in some models as not all patients had a sibling included in the study. To evaluate the possible consequences of this, we fitted both the full model and the final model within the Bayesian framework as well by means of a sensitivity analysis. These results (Supplementary Table S3) were comparable with the results presented in Table 2. Fifth, as described in the results, 143 out of 184 MRIs were successful. Attributing the reasons for missingness primarily to technical aspects, we assume that these missing data are random and not dependent on Lp(a) and/or cPWV values. Finally, although full validation of the 4D flow measurement method remains an objective for future research, the demonstrated strength of the underlying methodology, using the wavelet method, provides strong evidence for the reliability of our measured cPWV. Therefore, we are confident in the interpretation of our results within this clinical context.

### Clinical Implications

At this moment, no therapy is available to reduce Lp(a) levels. However, promising RNA- based therapies in secondary prevention settings are tested in adults (phase III) and seem to diminish Lp(a) levels drastically. As a result of these developments, the question might rise whether these drugs should also be tested in young patients with elevated Lp(a) levels as primary prevention care. Given that adult studies suggest that Lp(a) increases the risk of atherosclerosis even further in patients with FH and as they are already at increased CVD risk, this group might be a potential target population. However, before arriving at this juncture, establishing if high Lp(a) levels lead to atherosclerosis at a young age is of great importance. Although we did not observe a significant association between Lp(a) and arterial stiffness, studies with other surrogate markers of atherosclerosis do suggest that Lp(a) might contribute to other mechanisms of atherosclerosis, such as arterial wall thickening or endothelial dysfunction. Piechocki et al. (2024) suggest that atherosclerosis in FH predominantly affects central arteries, particularly the coronary arteries, due to lifelong exposure to elevated LDL-C [34]. Based on this, it could be argued that a central assessment of arterial stiffness, such as aortic or carotid-femoral PWV, might be more feasible to demonstrate an association between arterial stiffness and Lp(a) in these patients, should such an association truly exist. However, the relationship between local Lp(a)-related vascular changes and carotid alterations in adults with FH requires further investigation. Given the additional risk of Lp(a) in FH patients, measuring Lp(a) is recommended in all patients with FH to allow the identification of those patients at the highest CVD risk [35]. Despite the fact that no treatment for lowering Lp(a) treatment is available for these high-risk patients, it is of key importance to optimize other cardiovascular risk factors, such as not (start) smoking, and promote a healthy lifestyle from a young age.

## 5. Conclusions

This study provides unique and novel insights into the role of Lp(a) in arterial stiffness, using an advanced imaging technique, the 4D flow MRI, to assess cPWV as a marker for arterial stiffness. Our findings suggest that Lp(a) did not contribute statistically significantly to arterial stiffness. The lack of a statistically significant association may be attributed to unmeasured confounding factors in this observational study, as well as the narrow age range (and cPWV range) within a relatively young study population. However, it could also be argued that Lp(a) does not induce arterial stiffness in young adults and causes atherosclerosis by mechanisms beyond arterial stiffness at this age, such as endothelial dysfunction, measured by FMD, and increasing arterial wall thickening, measured by cIMT. Given the promising results of Lp(a)-lowering therapy in adults, there is a growing unmet need for a better understanding of the Lp(a)-mediated contribution to atherosclerosis in young FH patients. Based on our study, other surrogate markers of (early) atherosclerosis, such as cIMT and FMD, may be more suitable to fill this gap in our knowledge. To further explore the influence of Lp(a) on arterial stiffness in young patients who may be eligible for Lp(a)-lowering therapy in the future, larger prospective studies in this population using alternative markers, are urgently needed.

## Figures and Tables

**Table 1:** Demographic and clinical characteristics of the study population.

	Study Population N = 143
Male sex—no. (%)	69 (48.3)
Age (years)—mean (SD)	31.8 (3.2)
Body mass index (kg/m^2^)—mean (SD)	25.6 (4.2)
Systolic blood pressure (mmHg)—mean (SD)	121.2 (12.5)
Diastolic blood pressure (mmHg)—mean (SD)	74.1 (8.3)
Mean arterial pressure (mmHg)—mean (SD)	89.8 (8.8)
*Risk factors—no. (%)* Diabetes Hypertension Current smoking	1 (0.7) 13 (9.1) 33 (23.1)
*Lipids and lipoproteins* Total cholesterol (mmol/L)—mean (SD) HDL cholesterol (mmol/L)—mean (SD) LDL cholesterol (mmol/L)—mean (SD) LDL_cor30%_ cholesterol ^a^ (mmol/L)—mean (SD) LDL cholesterol goal ^b^—no. (%) Triglycerides (mmol/L)—median (IQR) Lipoprotein(a) (mg/dL)—median (IQR) Lipoprotein(a) levels >50 mg/dL—no. (%)	5.9 (1.8) 1.4 (0.4) 4.1 (1.7) 3.9 (1.8) 28 (19.6) 0.9 (0.6–1.2) 12.8 (5.4–24.4) 22 (15.4)
Age start statin use (years)—mean (SD)	14.0 (3.32)
Statin use—no. (%)	115 (80.4)
Pulse wave velocity—median (IQR)	1.62 (1.31–2.06)

^a^ Low-density lipoprotein cholesterol corrected for lipoprotein(a) cholesterol (lipoprotein(a) cholesterol was estimated as 30% of the lipoprotein(a) mass; ^b^ LDL-C goal defined as <2.6 mmol/L (corresponding to 100 mg/dL) HDL: high-density lipoprotein; IQR: interquartile range; LDL: low-density lipoprotein; mg/dL: milligram per deciliter; mmol/L: millimoles per liter; no: number; SD: standard deviation.

**Table 2:** Results of the mixed model analysis of the association between different patient characteristics and mean carotid PWV (univariable model) and the analyses adjusted for potential confounders (multivariable: full and final model).

	Univariable Model		Full Model *		Final Model ^#^	
	Beta (95% CI)	*p*-Value	Beta (95% CI)	*p*-Value	Beta (95% CI)	*p*-Value
Lipoprotein(a) (mg/dL)	−0.0014 (−0.0052 to 0.0023)	0.455	−0.0005 (−0.0043 to 0.0033)	0.782	−0.0005 (−0.0042 to 0.0032)	0.785

* Adjusted for age, sex, body mass index, low-density lipoprotein cholesterol corrected for lipoprotein(a) cholesterol (lipoprotein(a) cholesterol was estimated as 30% of the lipoprotein(a) mass), triglycerides, high-density lipoprotein cholesterol; ^#^ Adjusted for age, sex, body mass index, low-density lipoprotein cholesterol corrected for lipoprotein(a) cholesterol, mean arterial pressure. Mg/dL: milligrams per deciliter; 95% CI: 95% confidence interval.

## Data Availability

The data presented in this study are available on reasonable request from the corresponding author.

## Supplementary Materials

The following supporting information can be downloaded at: https://www.mdpi.com/article/10.3390/jcm14051611/s1, Table S1. Univariable association between patient characteristics and lipoprotein(a) (log transformed); Table S2. Results of the mixed models analysis of the association between different patient characteristics and mean carotid PWV (univariable model) and the analyses adjusted for potential confounders (multivariable: full and final model); Table S3. The posterior estimations of the multivariable association of the full and final model between patient characteristics and mean carotid PWV.
