# Supplementary material for: Association Between Lipoprotein(a) and Arterial Stiffness in Young Adults with Familial Hypercholesterolemia
Source: J Clin Med. 2025 Feb 27;14(5):1611. doi: 10.3390/jcm14051611 (PMC11901005; doi:10.3390/jcm14051611)
Supplement: Supplementary file 1 [file jcm-14-01611-s001.zip › jcm-3402593-supplementary.pdf]

Supplementary Materials

This appendix with supplemental material has been provided by the authors to give readers additional information about their work:

Association between lipoprotein(a) and arterial stiffness in young adults with familial hypercholesterolemia

by

Sibbeliene E. van den Bosch, Lotte M. de Boer, Alma Revers, Eric M. Schrauben, Pim van Ooij, Aart J. Nederveen, Willemijn E. Corpeleijn, John J.P. Kastelein, Albert Wiegman, Barbara A. Hutten

Contents

Supplementary Tables

	Page
**Table S1.** Univariable association between patient characteristics and lipoprotein(a) (log-transformed)	3
**Table S2.** Results of the mixed models analysis of the association between different patient characteristics and mean carotid PWV (univariable model) and the analyses adjusted for potential confounders (multivariable: full and a final model)	4
**Table S3.** The posterior estimations of the multivariable association of the full and final model between patient characteristics and mean carotid PWV	5

Table S1. Univariable association between patient characteristics and lipoprotein(a) (log-transformed)

	Univariable model	
	**Beta (95% CI)**	**p-value**
Age (years)	0.0242 (-0.0306 to 0.0790)	0.388
Female sex	0.085 (-0.243 to 0.4129)	0.613
Body mass index (kg/m^2)	0.0527 (0.0121 to 0.0932)	0.012
Mean arterial pressure (mmHg)	0.0079 (-0.0113 to 0.0271)	0.422
Smoking (yes vs. no)	-0.0301 (-0.4273 to 0.3670)	0.882
HDL cholesterol (mmol/L)	0.1055 (-0.3543 to 0.5652)	0.654
$LDL_{cor30\%}$ cholesterol[a] (mmol/L)	-0.0823 (-0.1764 to 0.0118)	0.089
Triglycerides (mmol/L)	-0.1527 (-0.3516 to 0.0461)	0.135
Statin use (yes vs. no)	0.0389 (-0.3844 to 0.4623)	0.857

[a] Low-density lipoprotein cholesterol corrected for lipoprotein(a) cholesterol (lipoprotein(a) cholesterol was estimated as 30% of the lipoprotein(a) mass

95% CI: 95% confidence interval; HDL: high-density lipoprotein; LDLcor30%: low-density lipoprotein corrected for Lp(a)-cholesterol; m: meter; mmol/L: millimoles per liter; kg: kilogram; vs.: versus

Table S2. Results of the mixed models analysis of the association between different patient characteristics and mean cPWV (univariable model) and the analyses adjusted for potential confounders (multivariable: full and final model)

	**Univariable model**		**Full model**		**Final model**	
	**Beta (95% CI)**	**p-value**	**Beta (95% CI)**	**p-value**	**Beta (95% CI)**	**p-value**
Lipoprotein(a) (mg/dL)	-0.0014 (-0.0052 to 0.0023)	0.455	-0.0005 (-0.0043 to 0.0033)	0.782	-0.0005 (-0.0042 to 0.0032)	0.785
Age (years)	-0.0140 (-0.0476 to 0.0197)	0.416	-0.0063 (-0.0402 to 0.0277)	0.697		
Female sex	-0.3311 (-0.5414 to -0.1207)	0.002	-0.2690 (-0.5159 to -0.0221)	0.029	-0.2793 (-0.4953 to -0.0632)	0.011
Body mass index (kg/m^2)	-0.0196 (-0.0451 to 0.0060)	0.136	-0.0263 (-0.0540 to -0.0014)	0.056	-0.0259 (-0.0526 to 0.0008)	0.055
Mean arterial pressure (mmHg)	0.0054 (-0.0070 to 0.0178)	0.392	0.00059 (-0.0084 to 0.0203)	0.406	0.0055 (-0.0077 to 0.0187)	0.408
Smoking (yes vs. no)	-0.0912 (-0.3485 to 0.1661)	0.488				
HDL cholesterol (mmol/L)	-0.2330 (-0.5278 to 0.0617)	0.124	-0.0084 (-03636 to 0.3468)	0.942		
LDL$_{cor30\%}$ cholesterol[a] (mmol/L)	0.0812 (0.0210 to 0.1414)	0.009	0.0599 (-0.0206 to 01403)	0.134	0.0690 (0.0081 to 0.1299)	0.025
Triglycerides (mmol/L)	0.0955 (-0.0321 to 0.2231)	0.145	0.0065 (-0.1347 to 0.1477)	0.924		
Statin use (yes vs. no)	-0.2131 (-0.4845 to 0.0584)	0.126	-0.0527 (-0.3949 to 0.2894)	0.747		

[a]Low-density lipoprotein cholesterol corrected for lipoprotein(a) cholesterol (lipoprotein(a) cholesterol was estimated as 30% of the lipoprotein(a) mass
95% CI: 95% confidence interval; HDL: high-density lipoprotein; LDL: low-density lipoprotein; m: meter; mg/dL: milligrams per deciliter; mmol/L: millimoles per liter; kg: kilogram; vs.: versus

Table S3. The posterior estimations of the multivariable association of the full and final model between patient characteristics and mean cPWV

	Full model<br>Beta (95% CI)	Final model<br>Beta (95% CI)
Lipoprotein(a) (mg/dL)	-0.0005<br>(-0.0038 to 0.0028)	-0.0005<br>(-0.0036 to 0.0026)
Age (years)	-0.0053<br>(-0.0352 to 0.0232)	
Female sex	-0.2767<br>(-0.4888 to -0.0642)	-0.2810<br>(-0.4627 to -0.1032)
Body mass index (kg/m^2)	-0.0267<br>(-0.0500 to -0.0036)	-0.0265<br>(-0.0486 to -0.0043)
HDL-cholesterol (mmol/L)	-0.0166<br>(-0.2917 to 0.3245)	
$LDL_{cor30\%}$ cholesterol[a] (mmol/L)	-0.0622<br>(-0.0067 to 0.1255)	0.0689<br>(0.0184 to 0.1222)
Triglycerides (mmol/L)	0.0080<br>(-0.1132 to 0.1291)	
Statin after 20 years	-0.0490<br>(-0.3375 to 0.2478)	
Mean arterial pressure (mmHg)	0.0062<br>(-0.0055 to 0.0186)	0.0057<br>(-0.0051 to 0.0166)

[a] Low-density lipoprotein cholesterol corrected for lipoprotein(a) cholesterol (lipoprotein(a) cholesterol was estimated as 30% of the lipoprotein(a) mass
95% CI: 95% confidence interval; HDL: high-density lipoprotein; LDL: low-density lipoprotein; m: meter; mg/dL: milligrams per deciliter mmol/L: millimoles per liter; kg: kilogram
